# A novel ferroptosis phenotype‐related clinical‐molecular prognostic signature for hepatocellular carcinoma

**DOI:** 10.1111/jcmm.16666

**Published:** 2021-06-04

**Authors:** Tuo Deng, Bingren Hu, Chen Jin, Yifan Tong, Jungang Zhao, Zhehao Shi, Tan Zhang, Liming Deng, Zhifu Sun, Gang Chen, Yi Wang

**Affiliations:** ^1^ Department of Hepatobiliary Surgery The First Affiliated Hospital of Wenzhou Medical University Wenzhou China; ^2^ Key Laboratory of Diagnosis and Treatment of Severe Hepato‐Pancreatic Diseases of Zhejiang Province The First Affiliated Hospital of Wenzhou Medical University Wenzhou China; ^3^ Department of Epidemiology and Biostatistics, School of Public Health and Management Wenzhou Medical University Wenzhou China; ^4^ Division of Biomedical Statistics and Informatics, Department of Health Sciences Research Mayo Clinic Rochester MN USA

**Keywords:** co‐expression network, ferroptosis, hepatocellular carcinoma, prognostic signature, risk‐stratification

## Abstract

Ferroptosis is a newly identified cell death mechanism and potential biomarker for hepatocellular carcinoma (HCC) therapy; however, its clinical relevance and underlying mechanism remain unclear. In this study, transcriptome and methylome data from 374 HCC cases were investigated for 41 ferroptosis‐related genes to identify ferroptosis activity‐associated subtypes. These subtypes were further investigated for associations with clinical and pathological variables, gene mutation landscapes, deregulated pathways and tumour microenvironmental immunity. A gene expression signature and predictive model were developed and validated using an additional 232 HCC cases from another independent cohort. Two distinct ferroptosis phenotypes (Ferroptosis‐H and Ferroptosis‐L) were identified according to ferroptosis gene expression and methylation in the patients with HCC. Patients with the Ferroptosis‐H had worse overall and disease‐specific survival, and the molecular subtypes were significantly associated with different clinical characteristics, mRNA expression patterns, tumour mutation profiles and microenvironmental immune status. Furthermore, a 15‐gene ferroptosis‐related prognostic model (FPM) for HCC was developed and validated which demonstrated accurate risk stratification ability. A nomogram included the FPM risk score, ECOG PS and hepatitis B status was developed for eventual clinical translation. Our results suggest that HCC subtypes defined by ferroptosis gene expression and methylation may be used to stratify patients for clinical decision‐making.

## 1 INTRODUCTION

Hepatocellular carcinoma (HCC) is a major type of primary liver cancer, accounting for ≥90% of all cases.^1^ Mortality associated with HCC remains the fourth leading cause of death among the common malignancies, and its incidence ranks fifth, with a consistently rising trend.^2^, ^3^ Although great strides have been made in HCC diagnosis and therapy, treatment options remain limited. Only early‐stage HCC patients with resectable tumours can undergo hepatectomy. For most HCC patients with unresectable tumours, sorafenib is considered as the only first‐line systemic chemotherapeutic drug.^4^, ^5^ However, high recurrence rates and drug resistance remain a problem for patients with HCC, with the prognosis still poor. Thus, the identification of novel therapeutic targets and development of a prognostic model to strategize for customized therapy are urgently needed for patients with HCC.

The hallmarks of cancer include cell death resistance ^6^ and numerous efforts have been made to employ programmed cell death in cancer therapy. Recently, a new way of programmed cell death involving iron‐dependent lipid peroxidation was defined as ferroptosis, which is genetically and biochemically distinct from other well‐understood forms of regulated cell death, such as apoptosis and programmed necrosis.^7^ Previous studies have identified that small‐molecules including erastin could induce ferroptosis by inhibiting system X_c_^‐^.^8^ Later on, other chemical compounds such as sorafenib were found to activate ferroptosis in HCC, which provided the potential for pharmaceutically inducing ferroptosis in patients with HCC.^9^, ^10^ Ferroptosis has been morphologically characterized by a series of mitochondrial changes, including small and condensed mitochondria, disappearance of the inner crista and outer membrane rupture of the mitochondria.^11^, ^12^ The molecular mechanisms underlying this lethal procedure include iron‐dependent lipid metabolism, which further causes an increase in reactive oxygen species and peroxidation products, as its name suggests.^13^ Similar to other cell death procedures, ferroptosis is mostly inhibited in tumour cells to maintain their survival. Therefore, modifying the ferroptosis status in patients with HCC may strongly affect their prognosis. Nevertheless, the correlation between ferroptosis and HCC prognosis remains obscure, and the underlying molecular mechanism remains unclear.

The present study identified two distinct ferroptosis phenotypes of HCC (Ferroptosis‐H and Ferroptosis‐L) based on the signature of mRNA expression and methylation of ferroptosis‐related genes. We compared the prognosis of these subgroups and found that Ferroptosis‐H had a worse prognosis. We further investigated the discrepancy in mRNA expression patterns, tumour mutation frequency and immune status. To elucidate the molecular mechanism of ferroptosis in HCC, we identified several prognosis defining genes and investigated their correlation with ferroptosis‐related genes. Furthermore, we developed a robust ferroptosis‐related prognosis prediction model and constructed a nomogram that may assist clinical decision‐making.

## 2 MATERIALS AND METHODS

### 2.1 Data acquisition and preprocessing

Using the TCGA data portal (https://portal.gdc.cancer.gov/), we downloaded a series of transcriptome data, methylation status data and mutation status data from 374 patients with HCC. The clinical information of these patients was also retrieved. Additional gene expression files from 232 HCC cases were extracted from the LIRI‐JP project in the International Cancer Genomics Consortium (ICGC; https://icgc.org/) database. All transcriptome data were Log_2_‐transformed for subsequent analysis. For the DNA methylation data, each CpG site was annotated and a beta‐value ranging from 0 to 1 was used to evaluate the methylation status of each CpG site. The average of CpGs in the promoter region of a gene was used to evaluate the methylation level of that gene. Samples that lacked important clinicopathological variables or survival information were excluded from the final analysis.

### 2.2 Clustering of ferroptosis‐related genes

Forty‐one ferroptosis‐related genes were identified using the Kyoto Encyclopedia of Genes and Genomes (KEGG; https://www.kegg.jp/). The promoter methylation status and mRNA expression levels of these 41 genes in patients with HCC from TCGA were screened for their association with overall survival (OS). Genes with statistically significant correlation between methylation status or mRNA expression level and OS (*P* < .05) were extracted for further clustering analysis to identify different ferroptosis phenotypes.

### 2.3 Differentially expressed gene identification and functional enrichment analysis

Each gene was measured for its adjusted *p*‐values between two ferroptosis phenotypes using the false‐discovery rate (FDR) method. Differentially expressed genes (DEGs) between two distinct ferroptosis phenotypes from TCGA were identified with the criteria FDR<0.05 and |log_2_fold‐change| >1 between two ferroptosis phenotypes. DEGs were further analysed for functional and pathway enrichment. To explore the potential mechanism and corresponding biomarkers underlying two ferroptosis phenotypes involved in HCC, the *c2.cp.kegg.v7.1.symbols.gmt* and *h.all.v7.0.symbols.gmt* annotated gene set files were selected as the reference gene set in the GSEA analysis and the threshold for significance was set at *P* < .05, with the FDR<0.25.

### 2.4 Tumour mutation analysis

By using the *maftool* package in R software,^14^ genes with the highest tumour mutation frequency (TMF) in patients with HCC from TCGA were analysed and visualized. We further performed at comparative analysis between two ferroptosis phenotypes in TCGA and identified genes with significantly different TMF (*P*‐value<.05).

### 2.5 Evaluation of immune status

The RNA‐seq data from TCGA were extracted to evaluate immune checkpoint‐related gene expression. Microenvironmental immune infiltration and stromal status were evaluated using the ESTIMATE algorithm, which provides a score of immune infiltration level, tumour purity and stromal content based on expression data.^15^ Based on the LM22 signature matrix, the CIBERSORT algorithm deconstructed the proportion of each human immune cell subtype in each patient.^16^ The proportion of 22 immune‐related cell types in different subtypes was visualized, and by running the Wilcoxon rank‐sum test, the difference in immune cell subtype proportions in the two ferroptosis‐related subtypes was inspected. Moreover, the ferroptosis‐specific gene expression patterns in individual tumour samples were transformed into a ferroptosis score using ssGSEA, and its relation with the proportion of each immune cell was determined by Spearman's test.

### 2.6 LASSO penalty regression and co‐expression network construction

We included the previously identified DEGs and significantly different TMF genes combined with the ferroptosis‐related genes defined by KEGG for further analysis. The LASSO‐logistic regression analysis was performed based on the expression level to identify key DEGs between the two ferroptosis phenotypes. Furthermore, by excluding the ferroptosis genes from key DEGs, we designated the remaining genes as prognosis defining genes. Moreover, we conducted a correlation test between the 41 ferroptosis‐related genes and the prognosis defining genes. The genes with an absolute value of correlation coefficient >0.3 and *P*‐value < .001 were plotted using Cytoscape (https://cytoscape.org/). The ferroptosis co‐expression network with *P*‐value <.001 and the absolute value of correlation coefficient >0.4 was illustrated using a Sankey diagram.

### 2.7 Construction and validation of a ferroptosis‐related prognostic model

The expression levels of the 41 ferroptosis‐related genes and previously identified prognosis defining genes with corresponding survival information were extracted from the TCGA and ICGC data sets for further analysis. However, the multivariat Cox regression analysis is unsuitable for highly correlated genes. To address this problem, the prognostic model in this study was constructed using the Lasso Cox regression analysis.^17^ By proportional size of genes, the shrinkage of the regression coefficient eliminated genes with zero regression coefficients and reserved genes with a nonzero weight. Finally, a ferroptosis‐related prognostic model (FPM) was developed. The risk score of FPM was calculated for each HCC patient in the TCGA and ICGC cohorts as the sum of the multiplied regression coefficient (β) by its expression level of genes retained in the FPM model [FPM risk score = ∑ EXP (mRNA) * β]. The cut‐off value of the FPM risk score for risk stratification was defined as the median value in TCGA. Patients with HCC in both TCGA and ICGC were stratified into high‐ or low‐risk subgroups separately. The log‐rank test was used to compare the OS between these two subgroups in each cohort. To assess the performance of the FPM in predicting OS, time‐dependent ROC was performed in each independent cohort.

### 2.8 Construction and evaluation of the nomogram

Univariate Cox regression was used to evaluate the association of clinicopathological variables as well as the FPM risk score with OS in the TCGA cohort. Variables with a *P*‐value < .001 were included in the downstream multivariate Cox regression analysis. The nomogram to predict the likelihood of OS was constructed by independent risk factors identified by multivariate Cox regression analysis (*P* < .05). The performance of the nomogram for predicting the OS was further evaluated by time‐dependent ROC method and calibration plots. The consistency of nomogram‐predicted probabilities and observed rates was evaluated using the concordance index (C‐index).

## 3 RESULTS

### 3.1 Ferroptosis‐related gene profiling identifies two HCC subtypes with different prognosis

**FIGURE 1:**
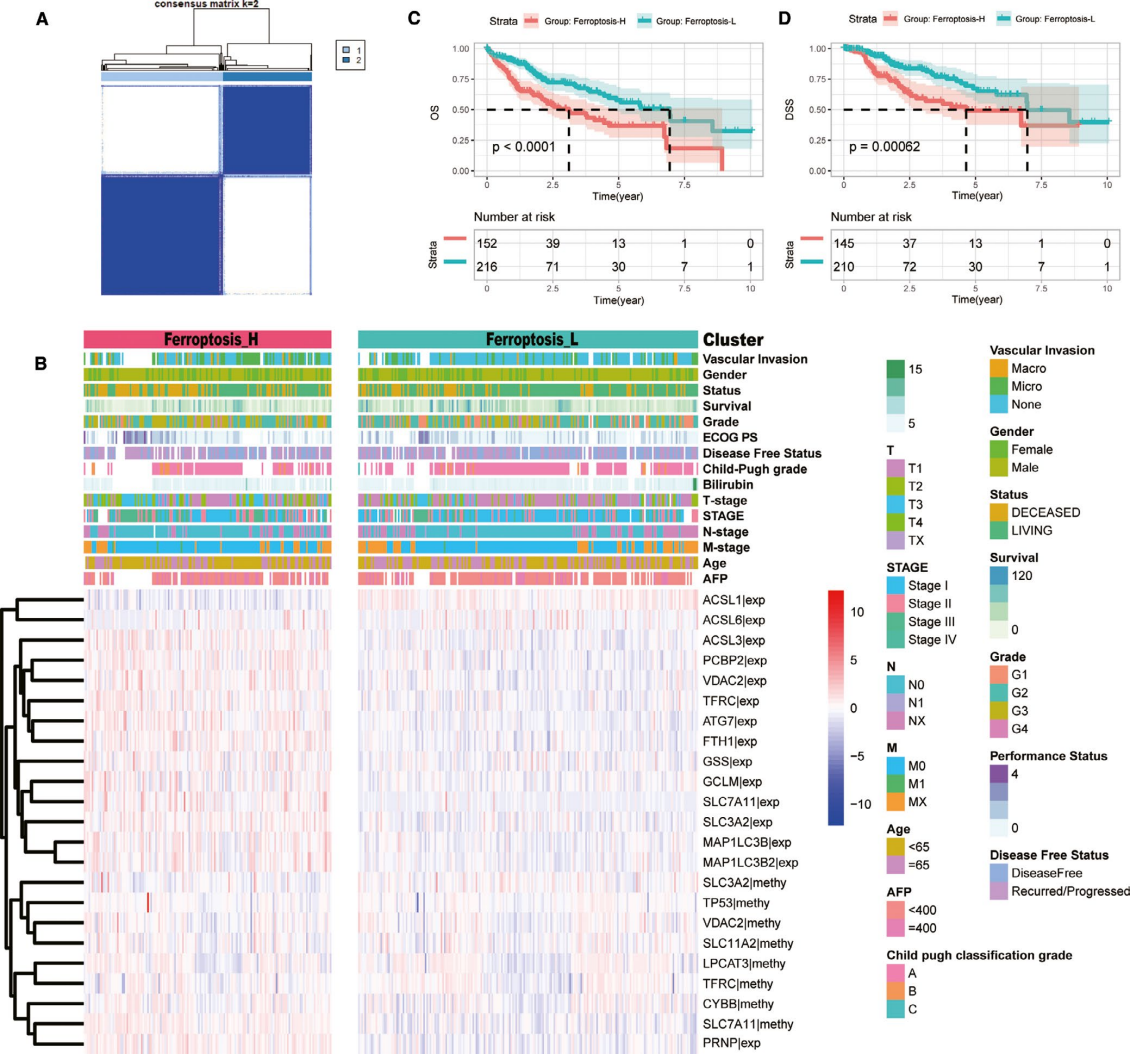
The landscape of ferroptosis‐related HCC subgroups in the TCGA cohort. (A) Two ferroptosis subgroups were generated via unsupervised consensus clustering. (B) Heatmaps of two ferroptosis‐related subgroups in the TCGA cohort. 15 mRNA expression level and 8 methylation level which were highly related to the prognosis of patients with HCC and used for clustering were illustrated. Clinicopathology characteristics were correspondingly listed. (C‐D) The survival time curve of two ferroptosis‐related subgroups. Ferroptosis‐H group had a worse OS (C) and DSS (D) than the Ferroptosis‐L group

Of the 41 ferroptosis‐related genes, 15 genes were identified for the expression level correlated with OS of HCC patients and 8 genes were screened out for the methylation status associated with OS of HCC patients (Figure S1). The unsupervised consensus cluster results showed that based on the gene expression level and methylation status, patients with HCC were divided into two distinct groups (Ferroptosis‐H and Ferroptosis‐L) with different ferroptosis phenotypes (Figure 1A,B).

The Kaplan‐Meier method was used to investigate the relationship between the ferroptosis phenotypes and prognosis of patients with HCC. The results showed that the Ferroptosis‐H phenotype had a worse OS than the Ferroptosis‐L phenotype (median OS: 3.11 vs. 6.93 years, *P* < .001) (Figure 1C). Moreover, we investigated the association between disease‐specific survival (DSS) and the ferroptosis phenotype. The results showed that the Ferroptosis‐H subgroup had poorer DSS than the Ferroptosis‐L subgroup (median DSS time: 4.63 vs. 6.96 years, *P* = .00062) (Figure 1D). These results indicated that the Ferroptosis‐H phenotype had a worse prognosis.

Furthermore, we compared the clinicopathological characteristics between the two ferroptosis subgroups and found that patients with HCC in the Ferroptosis‐H group had advanced T stage (*P* = .001), higher vascular invasion (*P* < .001), higher level of serum AFP (*P* = .015), and less differentiated tumours (*P* < .001) (Table 1, Figure 1B).

**TABLE 1:** Clinicopathological characteristics of HCC patients from the TCGA database

Clinicopathological variables	Total patients	Ferroptosis‐H	Ferroptosis‐L	*P*‐value
	(n = 363)	(n = 151)	(n = 212)	
Age (years)				.058
<65	213 (58.8)	97 (64.7)	116 (54.7)	
≥65	149 (41.2)	53 (35.3)	96 (45.3)	
Gender				.448
Female	117 (32.2)	52 (34.4)	65 (30.7)	
Male	246 (67.8)	99 (65.6)	147 (69.3)	
T‐stage				.001
T1 + T2	268 (74.2)	98 (64.9)	170 (81.0)	
T3 + T4	93 (25.8)	53 (35.1)	40 (19.0)	
N‐stage				.165
N0	246 (68.0)	108 (72.0)	138 (65.1)	
N1	116 (32.0)	42 (28.0)	74 (34.9)	
M‐stage				.106
M0	260 (71.6)	115 (76.2)	145 (68.4)	
M1	103 (28.4)	36 (23.8)	67 (31.6)	
AJCC stage				<.001
I + II	251 (74.0)	88 (63.3)	163 (81.5)	
III + IV	88 (26.0)	51 (36.7)	37 (18.5)	
AFP (mg/ml)				.015
<400	208 (76.5)	72 (68.6)	136 (81.4)	
≥400	64 (23.5)	33 (31.4)	31 (18.6)	
Child‐Pugh grade				.208
A	213 (90.6)	74 (87.1)	139 (92.7)	
B	21 (8.9)	11 (12.9)	10 (6.7)	
C	1 (0.4)	0 (0)	1 (0.7)	
ECOG Performance status				.030
0	162 (57.0)	53 (48.6)	109 (62.3)	
1	81 (28.5)	33 (30.3)	48 (27.4)	
2	26 (9.2)	13 (11.9)	13 (7.4)	
3	12 (4.2)	7 (6.4)	5 (2.9)	
4	3 (1.1)	3 (2.8)	0 (0)	
Family history of cancer				.131
NO	204 (65.2)	91 (70.0)	113 (61.7)	
YES	109 (34.8)	39 (30.0)	70 (38.3)	
Grade				<.001
G1‐2	224 (62.6)	72 (48.0)	152 (73.1)	
G3‐4	134 (37.4)	78 (52.0)	56 (26.9)	
Alcohol consumption				.848
NO	248 (68.3)	104 (68.9)	144 (67.9)	
YES	115 (31.7)	47 (31.1)	68 (32.1)	
Hepatitis B				.420
NO	266 (73.3)	114 (75.5)	152 (71.7)	
YES	97 (26.7)	37 (24.5)	60 (28.3)	
Hepatitis C				.128
NO	310 (85.4)	134 (88.7)	176 (83.0)	
YES	53 (14.6)	17 (11.3)	36 (17.0)	
Liver fibrosis Ishak score category				.067
No fibrosis	73 (34.4)	21 (27.6)	52 (38.2)	
Portal fibrosis	31 (14.6)	15 (19.7)	16 (11.8)	
Fibrous speta	28 (13.2)	12 (15.8)	16 (11.8)	
Nodular formation and incomplete cirrhosis	9 (4.2)	6 (7.9)	3 (2.2)	
Established cirrhosis	71 (33.5)	22 (28.9)	49 (36.0)	
Post‐operative radiotherapy				.089
NO	234 (98.3)	80 (96.4)	154 (99.4)	
YES	4 (1.7)	3 (3.6)	1 (0.6)	
Surgical margin resection status				.048
R0	319 (89.6)	127 (85.8)	192 (92.3)	
R1	37 (10.4)	21 (14.2)	16 (7.7)	
Vascular invasion				<.001
None	201 (65.5)	63 (52.1)	138 (74.2)	
Micro	90 (29.3)	47 (38.8)	43 (23.1)	
Macro	16 (5.2)	11 (9.1)	5 (2.7)	

### 3.2 Analysis of RNA‐seq data in HCC patients with different ferroptosis phenotypes

**FIGURE 2:**
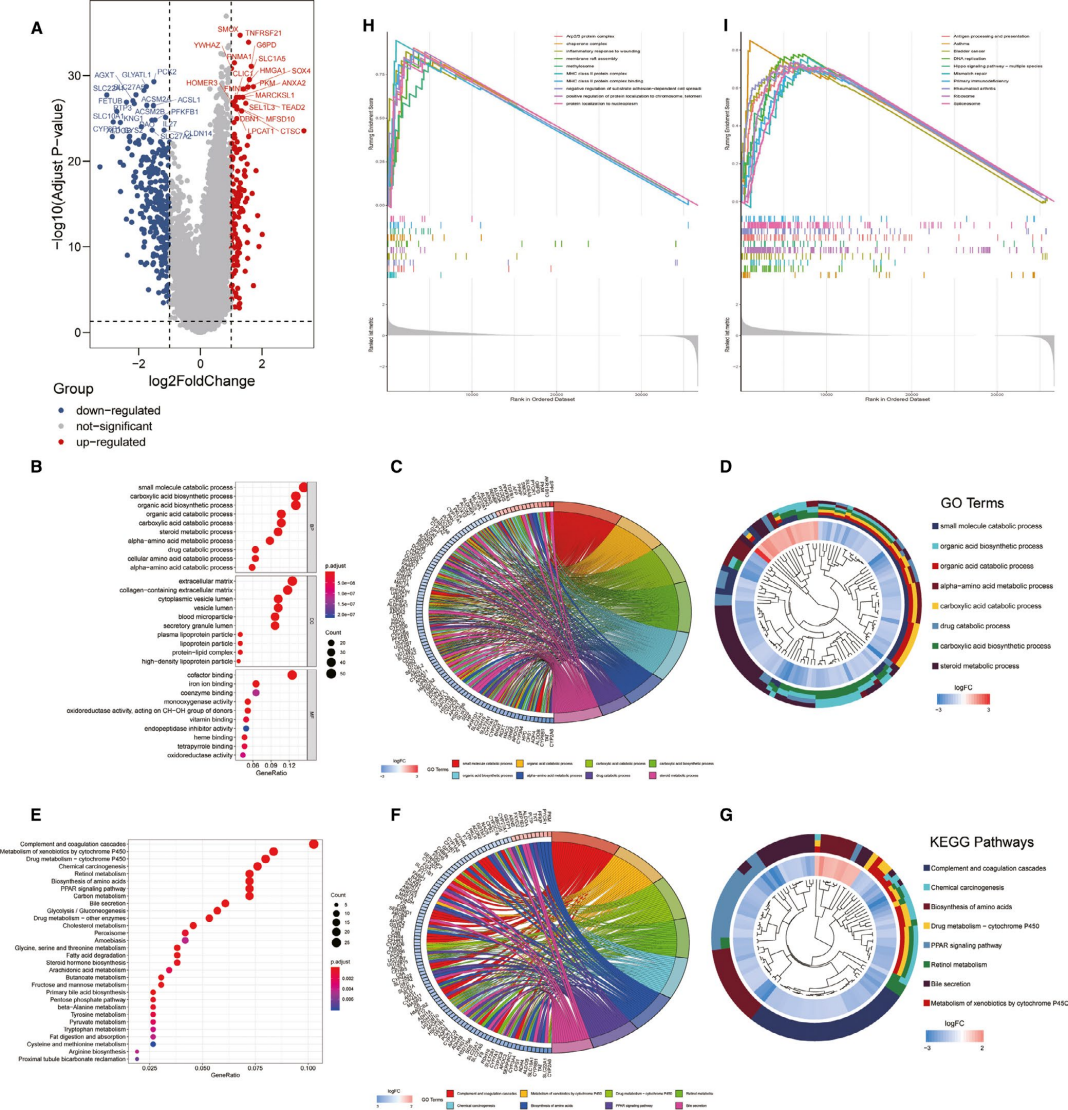
Differently expressed genes and enrichment analysis of two ferroptosis phenotype groups. (A) The volcano plot showed the significantly DEGs with FDR <0.05 and |log_2_FC| >1 between two ferroptosis phenotype. (B‐G) DEGs enrichment analysis. The bubble plot (B), circular plot (C) and cluster plot (D) of the biological process enriched for the DEGs between two ferroptosis phenotypes. The bubble plot (E), circular plot (F) and cluster plot (G) of KEGG pathways enriched for the DEGs between two ferroptosis phenotypes. (H‐I) The GSEA results for biological process (H) and KEGG pathways (I)

Transcriptome differential expression was performed between the newly identified Ferroptosis‐H and Ferroptosis‐L groups of HCC patients in the TCGA data set. At FDR < 0.05 and log2FC > 1, 416 genes were identified as DEGs, of which 150 genes were up‐regulated, and 266 genes were down‐regulated (Figure 2A). GO analysis revealed that these DEGs were mostly enriched in small molecule catabolic process, extracellular matrix and cofactor binding among BP, CC and MF, respectively (Figure 2B). The specific DEGs involved in GO terms are shown in Figure 2C,D. KEGG analysis demonstrated that these DEGs majorly participated in complement and coagulation cascades and cytochrome P450 associated metabolism (Figure 2E). The enriched pathways with involved DEGs and their expression levels were exhibited by circular and cluster plots (Figure 2F,G). GSEA was further performed to complement the results from KEGG and GO functions, and the results indicated that the enrichment in the inflammatory response to wounding, methylosome, MHC class II protein complex and binding as the top ones in biological processes. Antigen processing and presentation, the Hippo signalling pathway and mismatch repair were examples of KEGG pathway enrichment from GSEA (Figure 2H,I).

### 3.3 Analysis of mutations in HCC patients with different ferroptosis phenotypes

**FIGURE 3:**
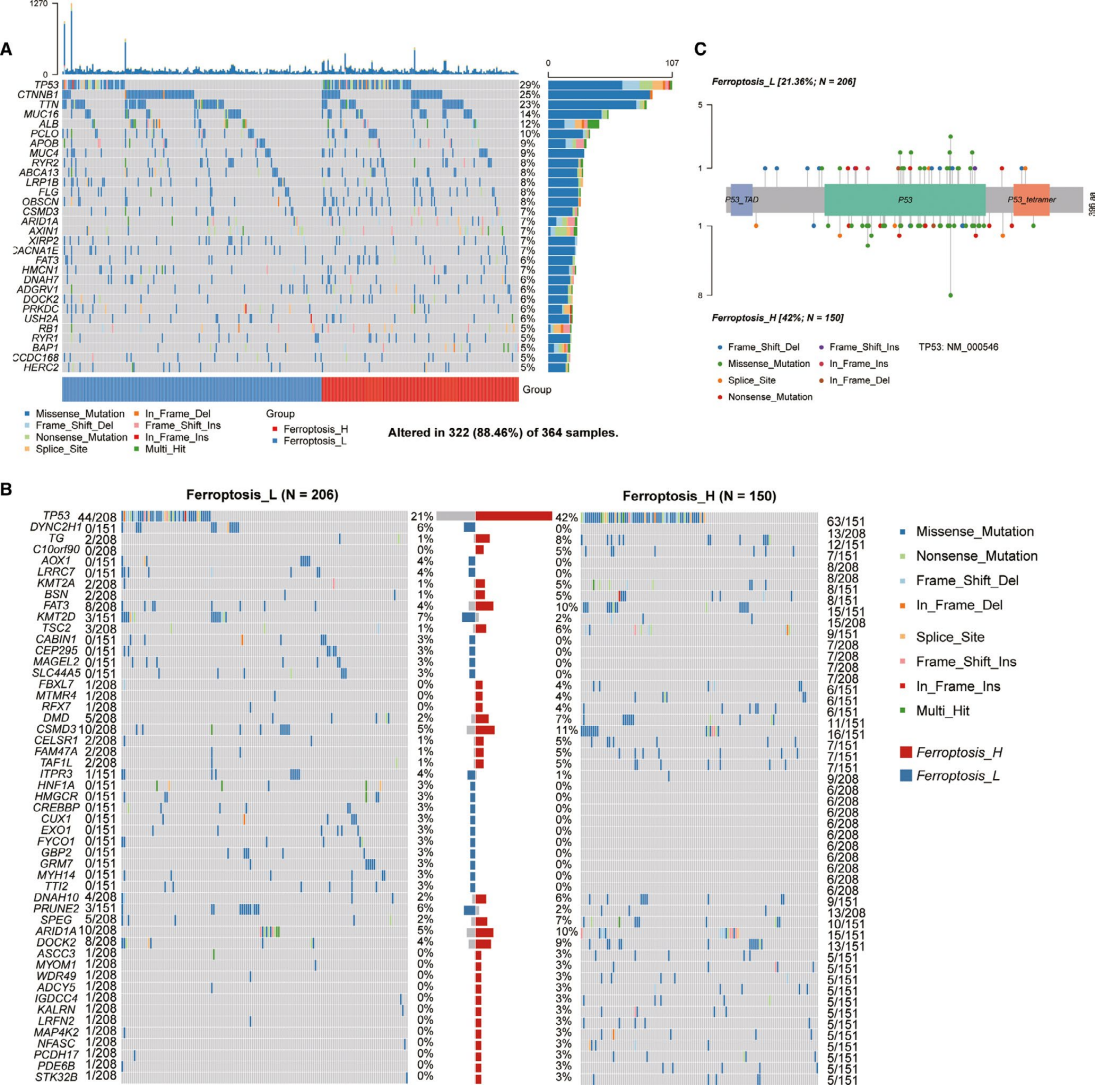
The mutation signature profile of HCC. The plots showed the TMB landscape in patients with HCC (A) and the correlation between these mutations (B). (C) Genes with the significantly differentially mutational burden in different ferroptosis‐related subgroup were showed by the waterfall plot, the central bar plot summarized the proportion of TMB of each gene in two different groups. (D) The lollipop plot of *TP53* gene showed the exact mutational position with type and its frequency

We illustrated the gene mutation patterns of HCC patients based on ferroptosis H and L status using exome‐seq data from TCGA with a waterfall plot, which illustrates genes with high mutation frequencies, mutation types and mutation distribution across genes in each patient (Figure 3A). Statistically, 51 genes were found to have different mutation frequencies (*P* < 05) between the two phenotypes (Figure 3B), among which *TP53* had the highest mutation frequency (29%) and the largest difference between the two phenotypes (42% in Ferroptosis‐H vs. 21% in Ferroptosis‐L). The location and rate of each mutation in *TP53* were displayed in the lollipop plot (Figure 3C). Besides *TP53*, other tumour suppressor genes such as *C10orf90*, *FAT3* and *TSC2* were identified as having significantly higher mutation rates in the Ferroptosis‐H group. These findings indicated that the poor prognosis of the Ferroptosis‐H group may result from the higher tumour mutation frequencies in the tumours.

### 3.4 The landscape of tumour immune status in patients with different ferroptosis phenotypes

**FIGURE 4:**
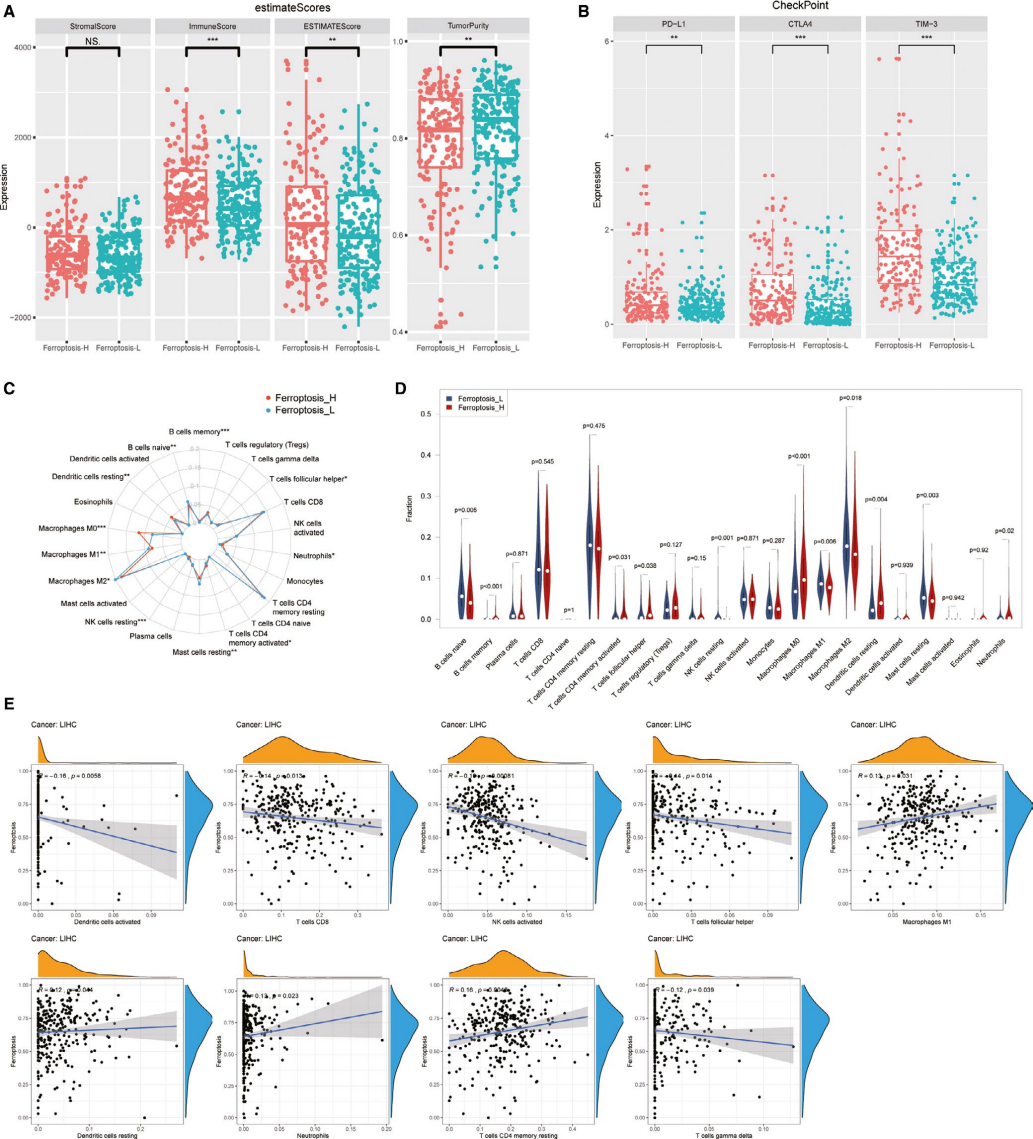
The immune landscape of two ferroptosis‐related subgroups. The ESTIMATE score (A) and immune checkpoints‐related gene expression (B) of two different ferroptosis phenotype groups. The proportional differences of 22 kinds of immune cells between patients with HCC were showed by radar plot (C) and violin plot (D). Scatter plots showed the significant correlation of tumour‐infiltrating immune cells proportion with ferroptosis score calculated by ssGSEA (*P* < .05) (E)

The microenvironmental immune and stromal status conducted by the ESTIMATE algorithm showed that the stromal status was not significantly different between the two groups, whereas the Ferroptosis‐H group had a higher immune score and lower tumour purity (Figure 4A). Targeted checkpoint immunotherapy provides a promising antitumor effect by eliminating the depressed immune response in the tumour microenvironment; thus, immune checkpoint‐related gene expression may serve as an indicator of the immunesuppressive status of a patient.^18^ *PD‐L1*, *CTLA‐4* and *TIM‐3* were immune checkpoint‐related genes, and their gene expression levels were significantly higher in the Ferroptosis‐H group than in the Ferroptosis‐L group (*P* < .01, .001 and .001, respectively) (Figure 4B).

To further explore the immune microenvironment differences between the two ferroptosis subgroups in patients with HCC, we investigated the proportions of 22 infiltrating immune cell subtypes in each patient using the CIBERSORT algorithm. The radar plot and violin plot summarized and displayed each tumour‐infiltrating immune cell proportion of the Ferroptosis‐H and Ferroptosis‐L groups (Figure 4C,D). The Ferroptosis‐H group had a significantly higher proportion of M0 type macrophages (*P* < .001), resting dendritic cells (*P* = .004), memory B cells (*P* < .001), neutrophils (*P* = .02), and active CD4‐positive memory T cells (*P* = .031); lower M1 (*P* = .006), M2 (*P* = .018) type of macrophages, resting mast cells (*P* = .003), naïve B cells (*P* = .005) and resting NK cells (*P* < .001) than the Ferroptosis‐L group. The ssGSEA score quantified enrichment levels of ferroptosis genes and analysed their relationship with the proportion of each immune cell type. Activated NK cells (*r* = −0.19, *P* = .031) and CD8‐positive T cells (*r* = −0.14, *P* = .013) were negatively correlated with ferroptosis level, whereas M1 macrophages (*r* = 0.13, *P* = .031) and CD4 (*r* = 0.16, *P* = .0048) were positively correlated with ferroptosis level (Figure 4E). These results suggest that the ferroptosis status of a tumour may affect its immune cell infiltration and immunity and the immune profile may guide the selection of immunotherapy.

### 3.5 Identification of the most distinctive genes that define ferroptosis‐related prognosis

**FIGURE 5:**
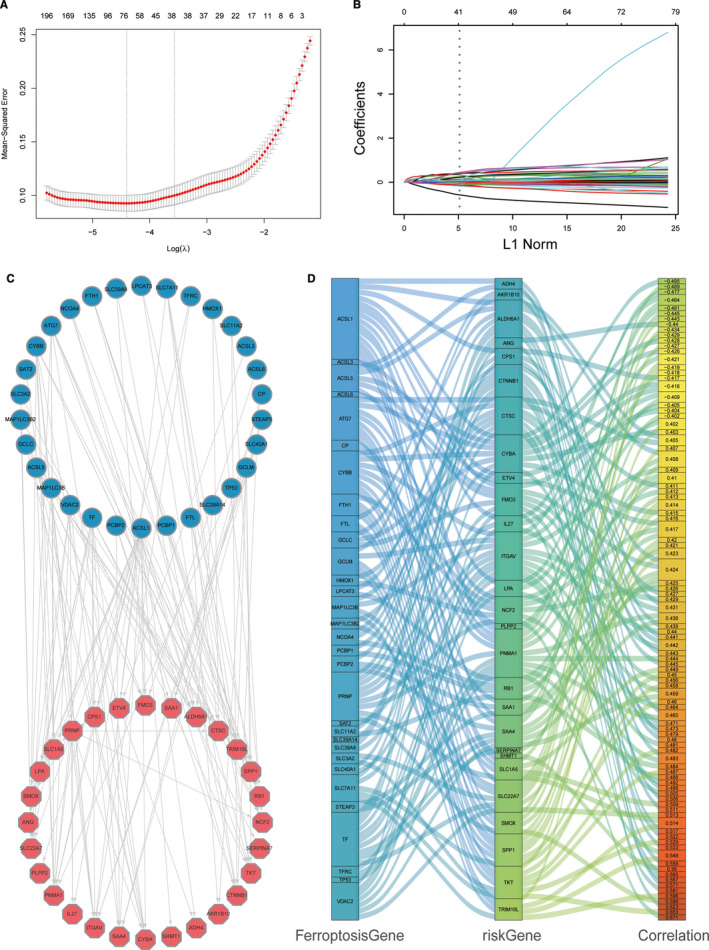
Identification of ferroptosis‐related risk gene and relationship with ferroptosis‐related gene. (A) Selecting the key differentially expressed genes related to the two ferroptosis subgroups in the LASSO model (λ). (B) LASSO coefficient spectrum of 508 genes enrolled and generate a coefficient distribution map for a logarithmic (λ) sequence. (C)The plot showed a significant correlation (*P* < .001) of the ferroptosis gene (left part) and risk gene (right part) with a correlation index higher than 0.3. (D) Sankey plot of significant correlation index ferroptosis gene (first column) and risk gene (second column) higher than 0.4 (*P* < .001)

To identify genes that best distinguished the Ferroptosis‐H and Ferroptosis‐L groups, we conducted Lasso logistic regression on 416 DEGs, 51 genes with different mutation frequencies, and 41 ferroptosis‐related genes from KEGG. Forty‐four genes were identified as key differentially expressed genes related to the two ferroptosis subgroups but 43 were not ferroptosis‐related genes as defined by KEGG (Figure 5A,B). We analysed the correlation between all the combinations of 41 ferroptosis‐related genes and the 43 ferroptosis prognosis defining genes and the combination pairs with significant associations (absolute correlation coefficient >0.3) were visualized where some pairs had up to 0.671 (Figure 5C). Moreover, we selected the combinations with an absolute correlation coefficient >0.4 and plotted their relationship with their specific correlation coefficient in the Sankey diagram (Figure 5D). The combination with an absolute correlation coefficient >0.4 is listed in Table S1.

### 3.6 Development and validation of the ferroptosis‐related prognostic model

**FIGURE 6:**
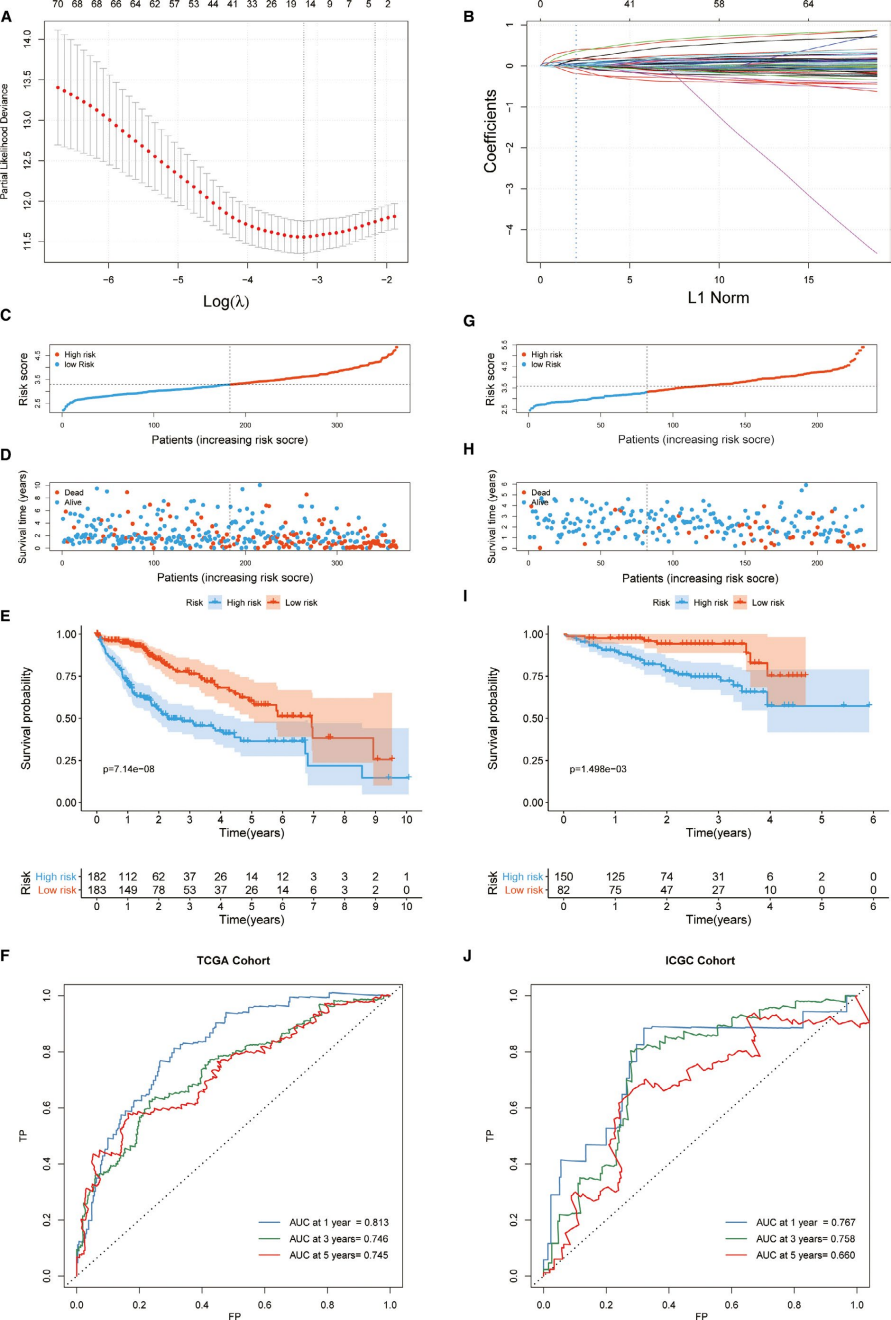
Construction and validation of FPM. (A) Selecting the best parameters for FPM in the LASSO model (λ). (B) LASSO coefficient spectrum of 84 genes enrolled and generate a coefficient distribution map for a logarithmic (λ) sequence. In the TCGA cohort, the FPM risk score of each patient was plotted and the high‐risk and low‐risk group were divided using median cut‐off value (C). The distribution of survival state (D), survival curve (E) and ROC curve (F) for the high‐risk and low‐risk group in the TCGA cohort were showed. The same cut‐off value of the FPM risk score was deployed in the ICGC cohort for risk stratification (G), and the survival state (H), survival curve (I) and ROC curve (J) of the high‐risk and low‐risk group in the ICGC cohort were showed

To develop a reliable predictive model for patient survival, we performed a Lasso penalized Cox regression analysis based on the mRNA expression level of 41 ferroptosis‐related genes combined with previously identified 43 ferroptosis phenotype defining genes in 374 patients with HCC in the TCGA cohort, and a 15‐gene signature model was established, named the FPM, which can best fit the prediction of OS in patients with HCC (Figure 6A,B). The regression coefficients of the genes used for the risk score calculation are listed in Table S2.

Based on the FPM risk score of each HCC patient, the TCGA cohort was divided into high‐ or low‐risk subgroups with the median value as the cut‐off level (cut‐off risk score = 3.2975). As shown in the KM curve, a significantly worse OS was found in the high‐risk subgroup than among its low‐risk counterparts (*P* < .001). We further evaluated the predictive performance of FPM using time‐dependent ROC curves. The AUC for 1‐, 3‐ and 5 years OS was 0.813, 0.746 and 0.745, respectively (Figure 6C‐F).

More importantly, we validated the robustness of FPM in the ICGC cohort with 232 patients with HCC. Using the cut‐off score retrieved from the TCGA cohort, the patients with HCC from the ICGC cohort were stratified into high‐risk (n = 140) and low‐risk (n = 92) groups. Consistent with the results in TCGA, the OS in the high‐risk group was significantly worse than that in the low‐risk group (*P* = .0015), and the AUC at 1‐, 3‐ and 5 years OS was 0.767, 0.758 and 0.660, respectively (Figure 6G‐J). The validation cohort demonstrated the robustness of FPM in predicting the prognosis of patients with HCC.

### 3.7 Establishment of a nomogram based on the model

**FIGURE 7:**
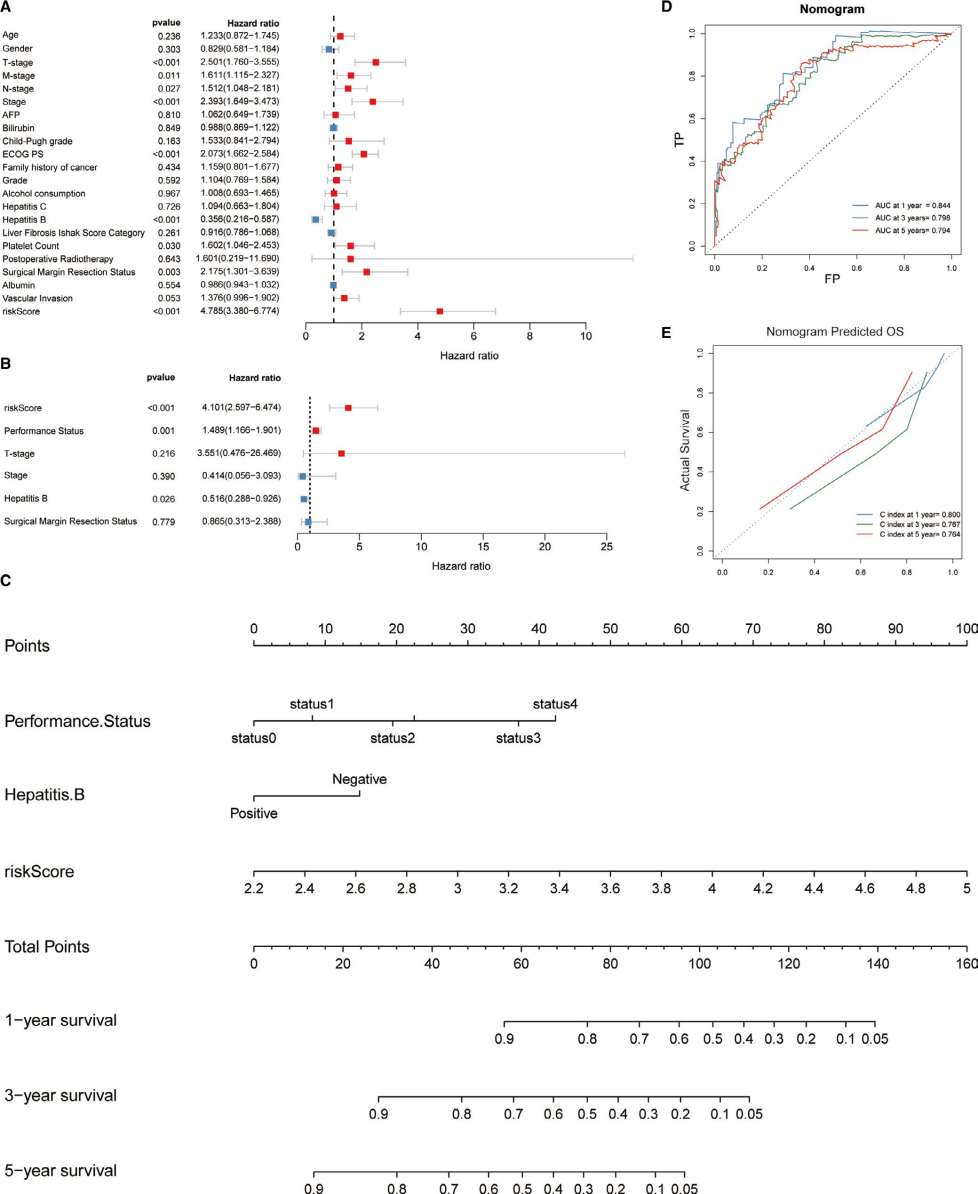
Establishment and assessment of the nomogram. Univariate (A) and multivariate (B) Cox regression analysis of the relationship between the FPM and clinicopathological characteristics regarding OS. (C) Nomogram constructed combined with ECOG PS and FPM. ROC (D) and calibration curve (E) of the nomogram for predicting the probability of 1‐, 3‐ and 5 years OS in the TCGA cohort

Univariate Cox regression analysis was performed to assess the association between OS and FPM risk score combined with clinicopathological variables (age, sex, family history of cancer, alcohol consumption, TNM classification, Child‐Pugh grade, ECOG performance status, vascular invasion status, surgical margin status, AFP, hepatitis B, hepatitis C, albumin, liver fibrosis Ishak score, platelet count and post‐operative radiotherapy) (Figure 7A). By including variables with a *P*‐value < .001 in further multivariate Cox regression analyses, the FPM risk score was shown to be an independent prognostic factor for OS, with a hazard ratio of 4.101 (95%CI: 2.597‐6.474; *P* < .001) (Figure 7B). We further constructed a nomogram with the FPM risk score, ECOG PS and hepatitis B. These factors were confirmed as independent prognostic factors through the multivariate Cox analysis (Figure 7C). The nomogram showed that the FPM risk score acted as the greatest and major weight factor in this scoring system. We further evaluated the predictive performance of this nomogram using the ROC curve and the result showed a higher AUC (1‐, 3‐ and 5 years OS was 0.842, 0.801 and 0.799, respectively) than the FPM risk score alone (Figure 7D). The calibration plots indicated that this nomogram had a strong agreement between predicted survival probabilities and actual observed outcome in which the C‐index at 1‐, 3‐ and 5 year OS was 0.798, 0.767 and 0.764, respectively (Figure 7E).

## 4 DISCUSSION

Successful evasion of regulated cell death is a major hallmark of cancer.^19^ Numerous efforts have been made to induce various kinds of programmed cell death in cancer to achieve therapeutic purposes.^20^, ^21^ The only targeted drug for HCC currently approved through clinical trials is sorafenib. It was designed as multiple tyrosine and downstream serine/threonine kinase inhibitor to inhibit proliferation and angiogenesis, as well as induce apoptosis. To date, only about 20% of advanced HCC patients may benefit from sorafenib treatment, and the OS of HCC patients have been unsatisfactorily extended by only 2.3 months.^4^ Recently, sorafenib was found to be an agonist in ferroptosisin a kinase inhibitory activity‐independent manner,^9^, ^10^ which has aroused wide interest in developing therapeutic strategies by targeting ferroptosis in HCC treatment. Numerous studies have investigated the underlying molecular mechanism of the ferroptosis pathway to elaborate its role in cancer biology. The results of previous studies demonstrated that the evasion of another type of cell death can cause sensitivity in ferroptosis.^22^ Thus, targeting ferroptosis provides promising and rational strategies to overcome drug resistance. ^23^, ^24^ However, the intrinsic ferroptosis status in patients with HCC and the mechanism by which ferroptosis status affects the prognosis of HCC remain largely unknown. Hence, a ferroptosis‐related model to categorize the risk of patients with HCC and investigate its molecular mechanism is urgently needed. For the first time, our study focused on patients with different prognosis‐related ferroptosis phenotypes in HCC and aimed to characterize its clinical utility and underlying molecular mechanism in different ferroptosis phenotypes. Our ferroptosis‐related gene clustering identified two HCC subtypes that were associated with different prognoses, as well as clinical and pathological features. We can infer that the Ferroptosis‐H group may have a higher degree of malignancy, invasive ability and tumour stage, resulting in its poor prognosis. To investigate the biological activity of ferroptosis in two ferroptosis phenotype subgroups, we screened the gene expression used in clustering. The primary molecular feature of ferroptosis was iron‐dependent induction of the Fenton reaction and NADPH‐dependent lipid peroxidation. Glutathione synthesis is considered a major way to eliminate reactive oxygen species (ROS) through glutathione peroxidase 4 (*GPX4*), thus suppressing the ferroptosis procedure.^25^, ^26^ Dixon et al demonstrated that the inhibition of system Xc‐, a member of the cystine/glutamate antiporter assembled by *SLC7A11* and *SLC3A2*, can cause the accumulation of ROS and thus induce ferroptosis.^27^, ^28^ Our heatmap showed that *SLC7A11* and *SLC3A2* were highly expressed in the Ferroptosis‐H group. Furthermore, *GCLM* and *GSS*, which are the first rate‐limiting enzymes of glutathione synthesis and the homodimer that catalyses the second step of glutathione biosynthesis, were also expressed at higher levels in the Ferroptosis‐H group. These results indicate that a higher glutathione level may suppress ferroptosis in the Ferroptosis‐H group and hence lead to a poor prognosis. Moreover, ferritin, transferrin and other proteins associated with iron metabolism play a vital role in preventing ferroptosis through their function in the storage of excess iron.^29^ Our heatmap also indicated an elevated expression in *FTH1*, which is a component of ferritin, and a slightly elevated *TFRC* that encoded the transferrin receptor. All of these results indicate that a more suppressed ferroptosis activation may occur in the Ferroptosis‐H subgroup than among its counterparts and thus may affect the clinical prognosis of patients with HCC.

Based on somatic mutation analysis, we characterized the mutation signature in the Ferroptosis‐H group with as having higher TMF of the tumour suppressor genes including *TP53*, *FAT3*, *TSC2* and *ARID1A,* with a significant difference. *TP53* is a tumour suppressor gene in many cancers and is largely involved in various types of cell death procedures including apoptosis, necrosis and autophagy.^25^, ^30^, ^31^ Activation of *TP53* was also found to be essential for the ferroptosis process through direct transcriptional inhibition of *SLC7A11*.^32^ Our results showed that the group with a higher mutation frequency on *TP53* was also related to a higher expression of *SLC7A11*. It is important to note that *SLC7A11* and *TP53* were part of the 15‐gene signature found to be highly predictive for the outcome of patients with HCC *ARID1A* is a commonly mutated gene in cancer and was reported as a suppressor of tumour initiation in liver cancer.^33^ *TSC2*, a tumour suppressor complex, is part of the tuberous sclerosis complex. The mutation of *TSC2* may induce the formation of tumours by affecting mTOR inhibition.^34^ *FAT3* is also a member of the FAT family involved in tumour suppression.^35^ Our analysis of tumour mutation frequency of different ferroptosis phenotypes suggested that a high mutation frequency in *TP53*, *ARID1A*, *TSC2* and *FAT3* in the Ferroptosis‐H phenotype might worsen the prognosis of patients with HCC by suppressing the ferroptosis process as well as the loss of tumour‐suppressive function in cancer.

In addition, we also investigated the landscape depicted by the immune score, combined with the ESTIMATE‐calculated tumour‐infiltrating immune cell proportions. Although the Ferroptosis‐H group had a higher immune infiltration state, the ESTIMATE analysis revealed a higher tumour‐infiltrated M0 macrophage combined with lower M1 and M2 macrophages in the Ferroptosis‐H group, which indicated a lower number of activated macrophages in the TME. A previous study indicated that activated CD4 + memory T cells can block CD8 + T cell activation and NK cell death, thus inducing an immunosuppressive effect.^36^, ^37^ Our results showed a higher proportion of CD4 + memory T cells in the Ferroptosis‐H group and a negative correlation between activated NK cells, CD8 + T cells and ferroptosis score, which may indicate an immunesuppressive state in the Ferroptosis‐H group. We further analysed PD‐L1 expression in two groups of HCC patients and found that the Ferroptosis‐H group had higher PD‐L1 expression, indicating an immunesuppressive state of TME, as reported by previous studies.^38^ All these suggest that the Ferroptosis‐H group may have a tumour‐suppressive state, which thus led to a poor prognosis.

After observing the poor prognosis in the Ferroptosis‐H group, we selected the key genes to represent genes with differential expression levels or with variant mutation frequency using the Lasso logistic method. Next, the correlation between 41 ferroptosis genes and 43 ferroptosis prognosis defining genes was analysed. These defining genes were differentially expressed in the ferroptosis‐related subgroups and were also closely related to ferroptosis‐related genes. However, their relationship with the ferroptosis pathway has not been investigated. They may interact with ferroptosis‐related genes and affect the prognosis of patients with HCC in an unrecognized manner. These findings warrant further investigation to untangle the complex interactions.

Next, FPM was developed based on 15 genes using the TCGA cohort to classify the prognosis of patients with HCC, and its robustness was verified using the ICGC cohort. The risk score may provide potential assistance for therapeutic decision‐making. Ferroptosis regulation treatment can be used to improve the prognosis of patients with HCC. Among the 15 genes used in the model, 8 were included in the ferroptosis‐related gene group, whereas 7 were not included; nevertheless, their potential mechanisms in affecting the prognosis of patients with HCC remain poorly understood. They may become a possible therapeutic target in further research.

Based on the FPM and significant clinical feature, a nomogram was constructed and validated. With a high consistency between nomogram‐predicted and actual 1‐, 3‐ and 5 years survival of patients with HCC, it may offer an easy‐to‐deploy prognostic prediction tool for clinicians.

Our study provides new insights into ferroptosis with regard to clinical prognostic and molecular changes of HCC through integrative multi‐omics analysis. Our results showed that ferroptosis genes can stratify patients with HCC into subgroups with different transcriptomes, mutations, immune patterns and especially different prognoses. Furthermore, a co‐expression network between the ferroptosis gene and other risk genes was built, which may guide further functional experiments involving the mechanism of ferroptosis and cancer biology. Finally, a robust model and nomogram were constructed and validated to predict the prognosis of patients with HCC. Nevertheless, our study has some limitations. First, our study had a retrospective design, and a more persuasive prospective study is needed to validate our results. Second, the molecular understanding based on functional experiments between ferroptosis‐related genes and risk genes should be validated to accelerate the understanding of the ferroptosis in the future. Finally, the model based on RNA‐seq may limit clinical application due to its high cost.

In summary, our team has established and validated a novel ferroptosis‐related 15‐gene prognosis prediction signature that allows robust stratification of prognoses among patients with HCC, while also facilitating the clinical identification of high‐risk HCC patients through the constructed nomogram.

## CONFLICT OF INTEREST

The authors declare that there is no conflict of interest.

## AUTHOR CONTRIBUTIONS

Tuo Deng: Formal analysis‐Equal, Writing—original draft‐Equal and Writing—review and editing‐Equal. Bingren Hu, Chen Jin, Yifan Tong, Jungang zhao, Zhehao Shi, Tan Zhang, Liming Deng and Zhifu Sun: Formal analysis‐Equal and Writing—review and editing‐Equal. Gang Chen: Conceptualization‐Equal, Data curation‐Equal and Writing—review and editing‐Equal. Yi Wang: Conceptualization‐Equal, Data curation‐Equal and Writing—review and editing‐Equal.

## Supporting information

Supplementary Material

## Data Availability

The data used to support the findings of this study are available from the corresponding author upon request.
