# Supplementary material for: A novel ferroptosis phenotype‐related clinical‐molecular prognostic signature for hepatocellular carcinoma
Source: J Cell Mol Med. 2021 Jun 4;25(14):6618–33. doi: 10.1111/jcmm.16666 (PMC8278110; doi:10.1111/jcmm.16666)
Supplement: Supplementary file 1 — Supplementary Material [file JCMM-25-6618-s001.docx]

Figure S1.

The correlation of OS of HCC patients with (**A**) 15 genes expression level and (**B**) 8 genes methylation level.


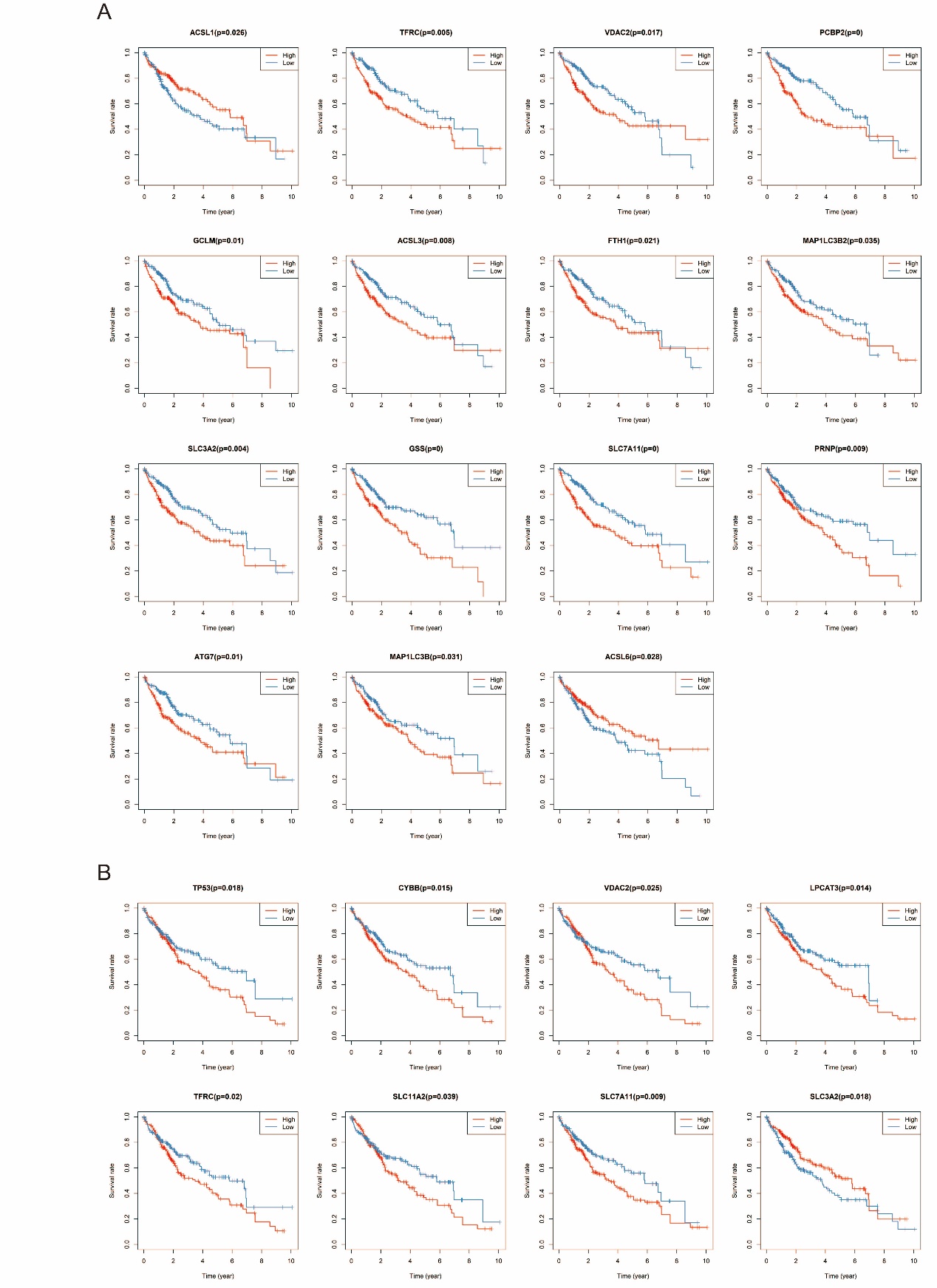


Table S1.

The correlation coefficient and *p*-value of each combination of ferroptosis gene and ferroptosis defining genes.

| **Ferroptosis Gene** | **Ferroptosis Defining Genes** | **Correlation Coefficient** | ***p-*value** |
| --- | --- | --- | --- |
| ACSL1 | CYBA | -0.4953 | 1.52E-24 |
| PCBP2 | SAA4 | -0.4891 | 6.88E-24 |
| TF | ITGAV | -0.4767 | 1.30E-22 |
| VDAC2 | IL27 | -0.4641 | 2.22E-21 |
| TF | PNMA1 | -0.4637 | 2.45E-21 |
| ACSL1 | PLPP2 | -0.4614 | 4.06E-21 |
| VDAC2 | SAA4 | -0.4454 | 1.26E-19 |
| MAP1LC3B | SLC22A7 | -0.4431 | 2.05E-19 |
| VDAC2 | ANG | -0.4402 | 3.73E-19 |
| ACSL1 | SMOX | -0.4339 | 1.34E-18 |
| ACSL1 | PNMA1 | -0.4286 | 3.82E-18 |
| ACSL5 | CYBA | -0.4282 | 4.12E-18 |
| VDAC2 | SLC22A7 | -0.4266 | 5.64E-18 |
| GCLC | CYBA | -0.4258 | 6.61E-18 |
| VDAC2 | LPA | -0.4213 | 1.60E-17 |
| MAP1LC3B2 | SLC22A7 | -0.4206 | 1.82E-17 |
| SLC3A2 | SLC22A7 | -0.4193 | 2.35E-17 |
| ACSL1 | SLC1A5 | -0.4177 | 3.15E-17 |
| PRNP | CPS1 | -0.4167 | 3.84E-17 |
| TF | CYBA | -0.4159 | 4.45E-17 |
| ACSL1 | ETV4 | -0.4157 | 4.67E-17 |
| ACSL5 | PNMA1 | -0.4090 | 1.62E-16 |
| PRNP | IL27 | -0.4087 | 1.73E-16 |
| SAT2 | ITGAV | -0.4046 | 3.65E-16 |
| VDAC2 | FMO3 | -0.4037 | 4.28E-16 |
| PCBP2 | SAA1 | -0.4023 | 5.59E-16 |
| CYBB | SMOX | 0.4019 | 5.96E-16 |
| TF | LPA | 0.4019 | 5.92E-16 |
| ATG7 | CYBA | 0.4031 | 4.77E-16 |
| NCOA4 | ALDH6A1 | 0.4050 | 3.37E-16 |
| PRNP | CTSC | 0.4054 | 3.16E-16 |
| FTH1 | TRIM16L | 0.4070 | 2.36E-16 |
| SLC39A8 | ALDH6A1 | 0.4078 | 2.03E-16 |
| ATG7 | SPP1 | 0.4083 | 1.86E-16 |
| LPCAT3 | RB1 | 0.4085 | 1.79E-16 |
| SLC7A11 | ITGAV | 0.4095 | 1.49E-16 |
| NCOA4 | RB1 | 0.4096 | 1.45E-16 |
| TFRC | PNMA1 | 0.4099 | 1.38E-16 |
| HMOX1 | CTSC | 0.4111 | 1.11E-16 |
| PRNP | NCF2 | 0.4116 | 1.01E-16 |
| TF | SERPINA7 | 0.4127 | 8.13E-17 |
| ATG7 | TKT | 0.4138 | 6.60E-17 |
| CYBB | SPP1 | 0.4142 | 6.18E-17 |
| PRNP | CYBA | 0.4153 | 5.01E-17 |
| ACSL1 | SLC22A7 | 0.4160 | 4.37E-17 |
| FTH1 | NCF2 | 0.4168 | 3.78E-17 |
| SLC11A2 | RB1 | 0.4171 | 3.59E-17 |
| ACSL3 | ETV4 | 0.4171 | 3.55E-17 |
| ATG7 | SMOX | 0.4199 | 2.07E-17 |
| FTH1 | SPP1 | 0.4213 | 1.59E-17 |
| TF | SAA4 | 0.4232 | 1.11E-17 |
| MAP1LC3B | SPP1 | 0.4232 | 1.10E-17 |
| ACSL6 | FMO3 | 0.4235 | 1.03E-17 |
| TF | CPS1 | 0.4238 | 9.78E-18 |
| CP | SAA1 | 0.4239 | 9.67E-18 |
| STEAP3 | SAA4 | 0.4240 | 9.48E-18 |
| STEAP3 | SAA1 | 0.4247 | 8.18E-18 |
| HMOX1 | NCF2 | 0.4262 | 6.10E-18 |
| PCBP2 | CTNNB1 | 0.4273 | 4.99E-18 |
| SLC40A1 | ITGAV | 0.4293 | 3.31E-18 |
| SLC39A8 | FMO3 | 0.4306 | 2.59E-18 |
| GCLM | AKR1B10 | 0.4310 | 2.36E-18 |
| CYBB | PRNP | 0.4356 | 9.38E-19 |
| CP | SAA4 | 0.4357 | 9.32E-19 |
| TP53 | PNMA1 | 0.4362 | 8.33E-19 |
| SLC39A14 | ALDH6A1 | 0.4387 | 5.04E-19 |
| MAP1LC3B | ITGAV | 0.4398 | 4.06E-19 |
| TF | ALDH6A1 | 0.4409 | 3.20E-19 |
| GCLC | FMO3 | 0.4415 | 2.83E-19 |
| SLC7A11 | CTSC | 0.4420 | 2.55E-19 |
| ACSL1 | CPS1 | 0.4432 | 2.01E-19 |
| VDAC2 | PNMA1 | 0.4442 | 1.62E-19 |
| MAP1LC3B2 | CTSC | 0.4446 | 1.49E-19 |
| PRNP | SLC1A5 | 0.4488 | 6.17E-20 |
| SLC7A11 | SPP1 | 0.4499 | 4.90E-20 |
| ACSL1 | IL27 | 0.4563 | 1.23E-20 |
| GCLM | TKT | 0.4578 | 8.88E-21 |
| PRNP | ITGAV | 0.4586 | 7.46E-21 |
| ACSL1 | SAA4 | 0.4595 | 6.25E-21 |
| GCLM | SPP1 | 0.4599 | 5.66E-21 |
| ACSL1 | LPA | 0.4642 | 2.21E-21 |
| CYBB | PNMA1 | 0.4647 | 1.98E-21 |
| ACSL5 | ADH4 | 0.4652 | 1.75E-21 |
| CYBB | CYBA | 0.4706 | 5.27E-22 |
| ACSL1 | FMO3 | 0.4731 | 2.92E-22 |
| FTL | AKR1B10 | 0.4795 | 6.77E-23 |
| SLC40A1 | CTNNB1 | 0.4795 | 6.69E-23 |
| TFRC | ITGAV | 0.4814 | 4.36E-23 |
| SLC3A2 | TKT | 0.4824 | 3.41E-23 |
| LPCAT3 | CTNNB1 | 0.4827 | 3.20E-23 |
| ATG7 | NCF2 | 0.4827 | 3.18E-23 |
| PCBP1 | RB1 | 0.4839 | 2.41E-23 |
| FTH1 | TKT | 0.4870 | 1.13E-23 |
| ATG7 | PNMA1 | 0.4885 | 8.05E-24 |
| GCLM | ITGAV | 0.4924 | 3.07E-24 |
| MAP1LC3B | CTSC | 0.4979 | 7.97E-25 |
| CYBB | ITGAV | 0.5054 | 1.22E-25 |
| GCLC | ALDH6A1 | 0.5055 | 1.18E-25 |
| ATG7 | SLC1A5 | 0.5087 | 5.26E-26 |
| ACSL1 | ADH4 | 0.5107 | 3.15E-26 |
| TF | SLC22A7 | 0.5130 | 1.71E-26 |
| SLC7A11 | TRIM16L | 0.5137 | 1.44E-26 |
| CYBB | SLC1A5 | 0.5140 | 1.33E-26 |
| ACSL5 | ALDH6A1 | 0.5170 | 5.95E-27 |
| SLC11A2 | CTNNB1 | 0.5219 | 1.61E-27 |
| SLC7A11 | TKT | 0.5290 | 2.36E-28 |
| TF | ANG | 0.5330 | 7.82E-29 |
| PRNP | PNMA1 | 0.5479 | 1.11E-30 |
| GCLM | TRIM16L | 0.5484 | 9.48E-31 |
| PCBP1 | CTNNB1 | 0.5577 | 5.87E-32 |
| ACSL1 | SHMT1 | 0.5598 | 3.09E-32 |
| NCOA4 | CTNNB1 | 0.5651 | 6.15E-33 |
| ATG7 | CTSC | 0.5675 | 2.91E-33 |
| ACSL1 | ALDH6A1 | 0.5706 | 1.09E-33 |
| ACSL5 | FMO3 | 0.5811 | 3.77E-35 |
| FTL | TRIM16L | 0.5955 | 2.87E-37 |
| PRNP | SMOX | 0.5983 | 1.10E-37 |
| CYBB | CTSC | 0.6233 | 1.22E-41 |
| FTL | TKT | 0.6535 | 6.46E-47 |
| CYBB | NCF2 | 0.6712 | 2.58E-50 |

Table S2.

The regression coefficients of each gene used in calculation of risk-score.

| **Gene** | **Regression Coefficients** |
| --- | --- |
| ADH4 | -0.006222131 |
| FMO3 | -0.007462769 |
| SAT1 | -0.067889742 |
| SPP1 | 0.009368088 |
| PCBP2 | 0.248663666 |
| GCLM | 0.070232583 |
| ACSL6 | -0.100918284 |
| MAP4K2 | 0.026276294 |
| HMOX1 | 0.077508874 |
| ATG5 | 0.042336183 |
| SLC7A11 | 0.093526323 |
| SMOX | 0.013124217 |
| TP53 | -0.139105236 |
| SLC1A5 | 0.102497469 |
| PRNP | 0.049516655 |
